# Chemostimuli for guanylyl cyclase-D-expressing olfactory sensory neurons promote the acquisition of preferences for foods adulterated with the rodenticide warfarin

**DOI:** 10.3389/fnins.2015.00262

**Published:** 2015-07-28

**Authors:** Kevin R. Kelliher, Steven D. Munger

**Affiliations:** ^1^College of Chiropractic, University of BridgeportBridgeport, CT, USA; ^2^Department of Pharmacology and Therapeutics, University of FloridaGainesville, FL, USA; ^3^Division of Endocrinology, Diabetes and Metabolism, Department of Medicine, University of FloridaGainesville, FL, USA; ^4^Center for Smell and Taste, University of FloridaGainesville, FL, USA

**Keywords:** uroguanylin, guanylin, GC-D, learning, mouse

## Abstract

Many animals have the ability to acquire food preferences from conspecifics via social signals. For example, the coincident detection of a food odor by canonical olfactory sensory neurons (OSNs) and agonists of the specialized OSNs expressing the receptor guanylyl cyclase GC-D (GC-D+ OSNs) will promote a preference in recipient rodents for similarly odored foods. It has been hypothesized that these preferences are acquired and maintained regardless of the palatability or quality of the food. We assessed whether mice could acquire and maintain preferences for food that had been adulterated with the anticoagulant rodenticide warfarin. After olfactory investigation of a saline droplet containing either cocoa (2%, w/w) or cinnamon (1%, w/w) along with a GC-D+ OSN-specific chemostimulus (either of the guanylin-family peptides uroguanylin and guanylin; 1–50 nM), C57BL/6J mice exhibited robust preferences for unadulterated food containing the demonstrated odor. The peptide-dependent preference was observed even when the food contained warfarin (0.025% w/w). Repeated ingestion of warfarin-containing food over four days did not disrupt the preference, even though mice were not re-exposed to the peptide stimulus. Surprisingly, mice continued to prefer warfarin-adulterated food containing the demonstrated odor when presented with a choice of warfarin-free food containing a novel odor. Our results indicate that olfactory-mediated food preferences can be acquired and maintained for warfarin-containing foods and suggest that guanylin peptides may be effective stimuli for promoting the ingestion of foods or other edibles with low palatability or potential toxicity.

## Introduction

Rodents use chemostimuli found on the breath and in urine and feces to communicate information about food (Galef, 2012). When a conspecific detects these semiochemicals simultaneously with a specific food odor it acquires a long-lasting preference for foods containing that same odor. These socially transmitted food preferences (STFPs) may result from the direct interaction of a novice rodent (observer) with an experienced one (demonstrator) (e.g., Galef et al., 1988; Crawley, 2007; Galef, 2012) or may be learned through pairing of the social odor with a particular feeding site (Arakawa et al., 2013). We have previously reported that both CS_2_ (found in rodent breath; Munger et al., 2010) and the guanylin family peptide uroguanylin (UG, a gut peptide excreted in urine and feces; Leinders-Zufall et al., 2007; Arakawa et al., 2013) mediate the formation of food preferences through activation of a specialized olfactory subsystem, the hallmark of which is a population of olfactory sensory neurons (OSNs) expressing the type D receptor guanylyl cyclase (GC-D). GC-D-expressing (GC-D+) OSNs detect both CS_2_ and guanylin-family peptides and are required for the acquisition of STFPs initiated by those semiochemicals.

Social interactions can lead to increased preferences to previously avoided foods in birds (Mason et al., 1984), rats (Galef et al., 1990), sheep (Thorhallsdottir et al., 1990), and cattle (Ralphs and Olsen, 1990). However, socially mediated flavor aversion has only been clearly shown to occur in birds (Mason et al., 1984); attempts in rats (Galef et al., 1990; Jing et al., 2014), sheep (Pfister and Price, 1996), and cattle (Cibils et al., 2008) have been unsuccessful in demonstrating a socially mediated avoidance of demonstrated foods or feeding sites. While chemostimuli that activate GC-D+ OSNs can promote the acquisition of preferences for laboratory chow containing innocuous flavorings such as cocoa or cinnamon (Galef et al., 1988; Munger et al., 2010; Arakawa et al., 2013), it is less clear whether these same mechanisms can promote preferences for foods containing toxic substances. In this study we investigated whether the guanylin-family peptides UG and guanylin, which specifically activate GC-D+ OSNs (Leinders-Zufall et al., 2007), can elicit a preference for odored food when that food has been tainted with the anticoagulant rodenticide warfarin. We further tested whether this preference was maintained over time or in the presence of unadulterated food. Together, our findings suggest that food preference acquisition mediated by guanylin-family peptides is robust, prolonged, and unaffected by aversive cues in foods.

## Materials and methods

### Animals

All experimental procedures were approved by the University of Maryland School of Medicine Institutional Animal Care and Use Committee. Mice were housed in an AAALAC accredited laboratory facility. Male C57BL/6J (B6) mice were obtained from the Jackson Laboratory (Bar harbor, MN). Mice were initially group housed (4–5 per cage) in standard cages (28 × 17 × 12.5 cm) with filter-top lids. All mice received water and standard rodent chow *ad libitum* prior to the experiments. The room in which the mice resided was environmentally controlled on a 12:12 h light:dark cycle (0600–1800 h lighting) at a temperature of 21°C, relative humidity of 50–60%.

### Food preference testing

Food preference assays were modified from those used previously for testing the social transmission of food preference in rats and mice (Galef et al., 1983; Posadas-Andrews and Roper, 1983; Valsecchi and Galef, 1989; Crawley, 2007; Ryan et al., 2008; Munger et al., 2010; Arakawa et al., 2013). In all experiments, subject mice were housed in groups of two or three for 3 days in standard cages with the food container placed on the cage floor. Mice were fed a crushed rodent diet (2018SX, Harlan) to habituate them to powdered food. The amount of food was restricted to 2 g/mouse/day to facilitate feeding during the food preference tests. Throughout the experiment mice were monitored for weight loss and overall health. Food deprived mice were removed from the experiment if their body weight dropped more than 25%. Mice fed food containing warfarin were also removed from the experiment and euthanized if they began showing significant signs of sickness. Food preference was quantified by computing the ratio of the demonstrated food consumed vs. the total food consumed by the subject mice (preference ratio, PR). All data were expressed as mean ± S.E.M. Differences were accepted as significant if *p* < 0.05 (see below for details of statistical tests).

#### Concentration-response to uroguanylin (UG) and guanylin (Experiment 1)

B6 mice were randomly assigned into three groups, with each group receiving different concentrations of UG. On the first test day, each mouse was moved to individual cages and presented with a petri dish containing a drop of saline (150 μl) with either cocoa (2%, Hershey's) or cinnamon (1%, McCormick) plus UG at a concentration of 50 nM (*n* = 8), 1 nM (*n* = 9), or 0 nM (*n* = 13). After 1 h of exposure [during which time mice would physically interact with the saline drop, allowing them to aspirate the peptide solution into the nasal cavity (Spehr et al., 2006)] mice were moved to clean cages. After 3 h, mice were presented with two food trays (3 g of food per tray): one odored with 1% cinnamon and one with 2% cocoa. Both trays were also adulterated with warfarin (0.025%, the concentration found in many commercial rodenticides). Food trays included a weighted base that captured spilled food (which was routinely minimal). After 1 h of feeding the food trays were removed and weighed to calculate the amount of food consumed. Experiments testing the efficacy of different concentrations of guanylin were performed identically to those testing UG except for the replacement of that peptide with guanylin at concentrations of 50 nM (*n* = 7), 1 nM (*n* = 7), or 0 nM (*n* = 13).

#### Duration of preference maintenance (Experiment 2)

B6 mice were randomly assigned into two groups, one to be exposed to saline containing UG (50 nM) plus odor (*n* = 8) and the other to saline plus odor alone (*n* = 8). Testing was done over a 5-day period. On Day 1 of this experiment we used the same testing paradigm as in Experiment 1 except that all mice were placed back into the home cages at the end of testing. On day 2 mice were transferred from their home cages to testing cages 3 h prior to testing. Mice were then given the choice to feed on warfarin-adulterated food odored with either cocoa or cinnamon. After 1 h trays were removed and weighed to determine the amount of each food consumed. Mice were placed back into their home cages until retesting on day 5. Mice were maintained on a restricted diet of powdered normal rat chow (2 g/mouse/day) during the non-testing days (days 3, 4). Mice typically exhibited signs of warfarin effects on later days, including lethargy, hunched posture and/or a dull coat, and were removed from the study if these signs were moderate or severe.

#### Preference maintenance given a novel odor choice (Experiment 3)

B6 mice were randomly assigned into three groups, with one group exposed to saline containing an odor (1% cinnamon or 2% cocoa) plus UG (50 nM; *n* = 10) and the second saline plus odor alone (*n* = 10). Testing was done over a 2-day period. On Day 1 of this experiment we used the same testing paradigm as in Experiment 1 except that all mice were placed back into the home cages at the end of testing. On Day 2, mice were transferred from their home cages to their testing cages 3 h prior to testing. Mice were then given the choice to feed on food containing the demonstrated odor and adulterated with warfarin (0.025%) or food containing a completely novel food odor (1% ginger) and no warfarin. After 1 h of feeding, food trays were removed and weighed to determine the amount of each food consumed.

### Data analysis

Food preferences were calculated as a ratio of demonstrated food consumed/ non-demonstrated food consumed, where the “demonstrated food” contained the demonstrated odor and the “non-demonstrated food” contained the novel odor. Data for Experiments 1 and 3 were each analyzed using a One-Way ANOVA followed by Tukeys *post-hoc* tests. Data for Experiment 2 was analyzed using a Two-Way, repeated measures ANOVA with presented odor and test day as the independent variables. Tukeys *post-hoc* tests were used for multiple comparisons of significant results.

## Results

### Nanomolar concentrations of guanylin-family peptides can induce preferences for warfarin-adulterated foods

**Figure 1:**
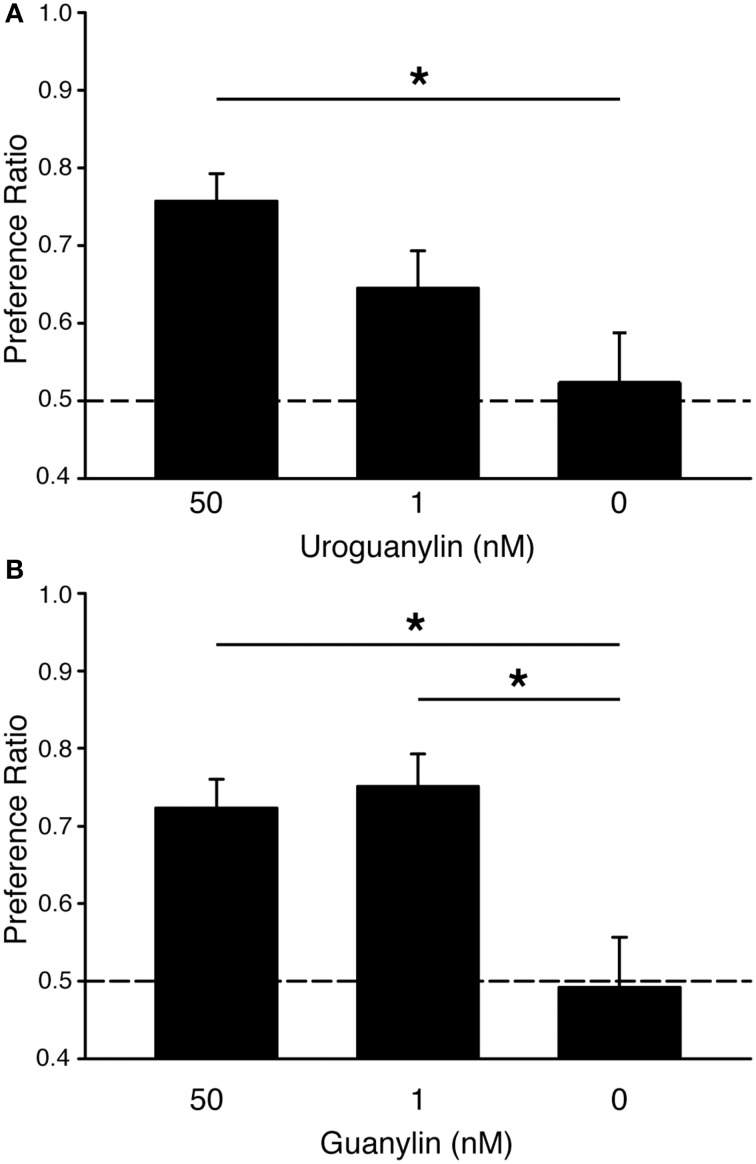
**Guanylin family peptides promote preferences for odored food adulterated with warfarin. (A)** C57BL/6J mice acquired preferences for food containing a demonstrated odor when that odor was paired with 50 nM uroguanylin (UG) even when both food choices had been adulterated with the rodenticide warfarin (0.025%). Mice were demonstrated a food odor paired with 50 nM UG (*n* = 8), 1 nM UG (*n* = 9), or 0 nM UG (*n* = 13). One-Way ANOVA [*F*_(1, 29)_ = 4.31, *P* < 0.05] followed by Tukeys *post-hoc* test (^*^P < 0.05). **(B)** C57BL/6J mice acquired preferences for food containing a demonstrated odor when that odor was paired with either 50 nM or 1 nM guanylin even when both food choices had been adulterated with warfarin (0.025%). Mice were demonstrated a food odor paired with 50 nM guanylin (*n* = 7), 1 nM guanylin (*n* = 7), and 0 nM guanylin (*n* = 14). One-Way ANOVA [*F*_(1, 26)_ = 6.29, *P* < 0.01] followed by Tukeys *post-hoc* test (^*^P < 0.05). Dashed lines, no preference. Error bars, standard error of the mean.

We previously showed that mice form food preferences to odored food when the odor is first paired with uroguanylin (UG) at concentrations as low as 50 nM (Arakawa et al., 2013). However, electroolfactogram studies in the mouse olfactory epithelium showed significant responses at even lower concentrations of UG (EC_50_ < 1 nM) (Leinders-Zufall et al., 2007). Here, we found that mice exposed to a food odor plus 50 nM UG formed a preference for food adulterated with 0.025% warfarin and containing the demonstrated odor [PR = 0.76 ± 0.04 SEM; One-Way ANOVA: *F*_(1, 29)_ = 4.31, *p* < 0.05; Tukey's *post-hoc* vs. 0 nM UG control, *p* < 0.05] (Figure 1, Table 1). This preference was absent in mice exposed to food odor alone (PR = 0.52 ± 0.06 SEM). Mice exposed to food odor plus 1 nM UG exhibited a lesser preference (PR = 0.65 ± 0.05 SEM) that was not significantly greater than the 0 nM UG control (Tukeys's *post-hoc, p* > 0.05). Together, these results show that UG is similarly effective at eliciting a food preference in unadulterated (Arakawa et al., 2013) or warfarin-adulterated food (Figure 1).

**Table 1:** **Food consumed by observer mice in food preference assays (mean ± s.e.m.)**.

**Stimulus**		**Food consumed (g)**		
		**Total food**	**w/demonstrated odor**	**w/novel odor**
**EXPERIMENT 1**				
Odor alone		2.25 ± 0.10	1.15 ± 0.10	1.10 ± 0.11
Odor + UG (50 nM)		2.19 ± 0.20	1.62 ± 0.21	0.57 ± 0.12
Odor + UG (1 nM)		1.97 ± 0.15	1.37 ± 0.1	0.60 ± 0.09
**EXPERIMENT 2**				
Odor alone		2.89 ± 0.15	1.42 ± 0.11	1.47 ± 0.13
Odor + G (50 nM)		2.00 ± 0.25	1.47 ± 0.13	0.54 ± 0.09
Odor + G (1 nM)		2.10 ± 0.36	1.48 ± 0.2	0.7 ± 0.23
**EXPERIMENT 3**				
Odor alone	*Day 1*	2.19 ± 0.31	1.18 ± 0.31	1.01 ± 0.12
	*Day 2*	1.98 ± 0.31	0.94 ± 0.16	1.04 ± 0.17
	*Day 5*	0.55 ± 0.12	0.30 ± 0.08	0.25 ± 0.16
Odor + UG (50 nM)	*Day 1*	2.00 ± 0.25	1.47 ± 0.13	0.53 ± 0.09
	*Day 2*	2.10 ± 0.36	1.48 ± 0.2	0.62 ± 0.23
	*Day 5*	0.63 ± 0.06	0.04 ± 0.01	0.02 ± 0.01
**EXPERIMENT 4**				
Odor alone		2.21 ± 0.15	1.26 ± 0.14	0.95 ± 0.15
Odor + UG		2.51 ± 0.15	1.62 ± 0.12	0.89 ± 0.11
Odor alone w/novel choice		1.82 ± 0.22	0.96 ± 0.19	1.04 ± 0.19
Odor + UG w/novel choice		1.45 ± 0.19	1.15 ± 0.09	0.3 ± 0.18

*UG, uroguanylin; G, guanylin; Odor: either 2% cocoa or 1% cinnamon (counterbalanced)*.

Another guanylin-family peptide, guanylin, is the most effective chemostimulus (EC_50_ < 200 pM) yet identifed for GC-D+ OSNs (Leinders-Zufall et al., 2007). Here we observe that guanylin is also an effective social cue in the acquisition of food preferences in mice (Figure 1, Table 1). Mice showed significant preferences for food containing a demonstrated odor after exposure to that odor plus either 50 nM guanylin (PR = 0.72 ± 0.04 SEM) or 1 nM guanylin (PR = 0.75 ± 0.04 SEM) but not in controls containing odor only [PR = 0.49 ± 0.06 SEM; One-Way ANOVA, *F*_(1, 26)_ = 6.29, *p* < 0.01; Tukeys's *post-hoc* vs. 0 nM G control, *p* < 0.05].

### Mice maintain a preference for several days after a single uroguanylin exposure

**Figure 2:**
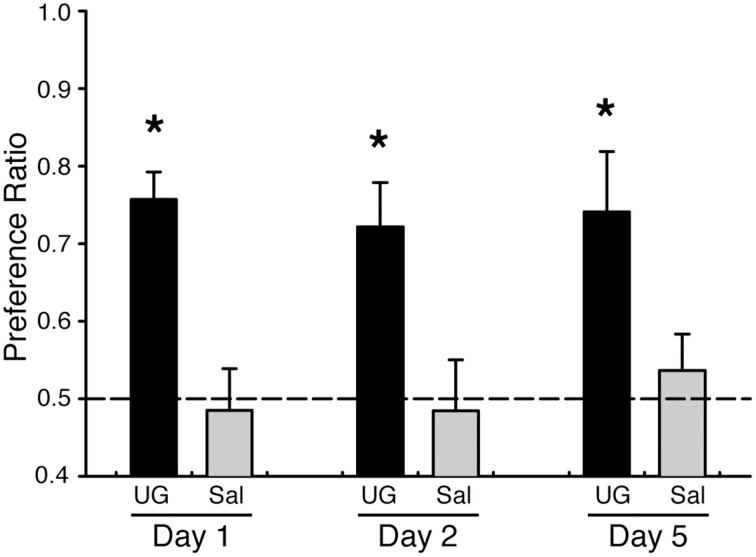
**Mice maintain UG-mediated food preferences even after ingestion of food adulterated with warfarin**. C57BL/6J mice were demonstrated a food odor paired with 50 nM UG (black, *n* = 8) or saline (gray, *n* = 8). Mice exposed to UG, but not saline controls, showed a significant preference for food containing the demonstrated odor and warfarin (0.025%); this preference was maintained after 5 days without additional exposure to UG. Two-Way repeated measures ANOVA on stimulus and day [*F*_(1, 46)_ = 19.55, *P* < 0.001] followed by Tukeys *post-hoc* test (^*^P < 0.01). Dashed lines, no preference. Error bars, standard error of the mean. One control mouse was removed from the study prior to Day 5 due to apparent significant distress.

**Figure 3:**
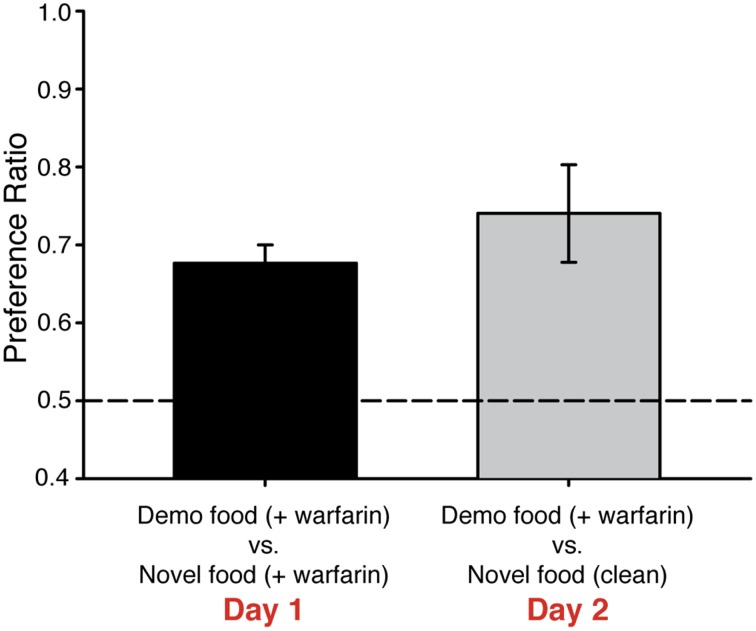
**Mice prefer demonstrated food containing warfarin to novel food without the rodenticide**. C57BL/6J mice (*n* = 10) were demonstrated a food odor paired with 50 nM UG. On Day 1, mice preferred food containing the demonstrated over food with a novel odor, although both foods contained warfarin (0.025%). On Day 2, these mice continued to prefer food containing both the demonstrated odor and warfarin even though the other food choice contained a novel odor but no warfarin. One-Way ANOVA [*F*_(1, 19)_ = 10.1, *P* < 0.005], followed by Tukeys *post-hoc* test (^*^P < 0.01). Dashed lines, no preference. Error bars, standard error of the mean.

Food preferences acquired after exposure to social cues can last for days or weeks. We tested whether preferences acquired after exposure to UG could be maintained for food adulterated with warfarin. As before, we found that mice exposed to a food odor plus 50 nM UG acquire a preference for food adulterated with 0.025% warfarin and containing the demonstrated odor (PR = 0.76 ± 0.04 SEM) when tested on the same day as the UG exposure; mice exposed to food odor alone did not demonstrate a preference (PR = 0.49 ± 0.05 SEM) (Figure 2, Table 1). These mice maintained a preference for the demonstrated odored food when retested 1 and 4 days after the UG exposure (Day 2: PR = 0.72 ± 0.06 SEM; Day 5: PR = 0.74 ± 0.08) despite having no additional exposure to UG (Figure 3). A Two-Way repeated measures ANOVA showed a significant effect of UG exposure [*F*_(1, 46)_ = 19.55, *p* < 0.001; Tukey's *post-hoc* vs. 0 nM UG, *p* < 0.01 on each test day] on the preference measures across the 5 day period.

### Mice maintain a preference for food containing warfarin even in the presence of unadulterated food

Warfarin can cause significant distress in rodents that ingest it even before reaching lethal levels. We next tested whether UG-dependent preferences for food adulterated with warfarin are maintained when mice are given a choice of unadulterated (i.e., warfarin-free) food that contains a novel odor. As before, mice were exposed to 50 nM UG plus a food odor or to food odor alone and then given a choice of foods containing warfarin and either the demonstrated or novel odor. These mice were then tested again the next day; however, on this second day of testing mice were given a choice of food containing warfarin plus the demonstrated odor or food containing a new odor (ginger) and no warfarin. Mice that had been exposed to UG maintained a strong preference for food containing the demonstrated odor [PR = 0.74 ± 0.06 SEM; One-Way ANOVA, *F*_(1, 19)_ = 10.1, *p* < 0.005] (Figure 3, Table 1).

## Discussion

The sensory cues that influence food choices include general odors, semiochemicals, tastes, and post-ingestive signals. In rodents, the social chemostimuli CS_2_, UG, and guanylin activate specialized GC-D+ OSNs to elicit the acquisition of food preferences (Leinders-Zufall et al., 2007; Munger et al., 2010; Zufall and Munger, 2010; Arakawa et al., 2013). Here, we found that UG and guanylin are highly effective stimuli for promoting the acquisition of preferences for foods, even if those foods contain the poison warfarin. These preferences remain robust over several days and are retained even when the mouse is given an alternative, unadulterated food choice.

GC-D+ OSNs are exquisitely sensitive to UG and guanylin, with responses seen upon stimulation with picomolar concentrations of the peptides (Leinders-Zufall et al., 2007); CS_2_ is also highly effective, activating these cells at submicromolar concentrations (Munger et al., 2010). Electroolfactrogram recordings from the main olfactory epithelium found guanylin (*K*_1/2_ = 66.1 pM) to be a somewhat more effective stimulus than UG (*K*_1/2_ = 247 pM) (Leinders-Zufall et al., 2007). Thus, we expected guanylin to be able to elicit a food preference at lower concentrations than UG. Indeed, this was the case. While 50 nM of either peptide was sufficient to elicit a significant preference in mice, only guanylin could do so at 1 nM (Figure 1). This sensitivity is reminiscent of other olfactory subsystems that couple the detection of semiochemicals to defined behavioral outputs. For example, threshold responses of vomeronasal sensory neurons to the exocrine gland-secreting peptides ESP1 (which elicits female sexual behaviors) and ESP22 (which inhibits male sexual behaviors) are each found in the low nanomolar range (Kimoto et al., 2007; Haga et al., 2010; Ferrero et al., 2013). OSNs expressing the trace amine-associate receptor TAAR4, which are important for predator avoidance, respond to carnivore odor β-phenylethylamine with an EC_50_ of ~1 pM (Zhang et al., 2013). The high value placed on the detection of semiochemicals may favor sensory cells that are exceedingly sensitive to specific stimuli.

The acquisition of STFPs involves the formation of short and long-term memories for food odors that can last weeks or possibly even months (Galef and Whiskin, 2003). There is good evidence that the acquisition and retrieval of short-term memories for STFPs requires the ventral hippocampus (Countryman et al., 2005; Ross and Eichenbaum, 2006). Damage to the ventral hippocampus 1–2 days after training eliminates memory for food odors, but retrieval is not inhibited if the lesion is performed 21 days after training (Ross and Eichenbaum, 2006). Long-term memory for STFPs involves consolidation of memories in neocortical areas and the amygdala (Smith et al., 2007). In our experiments we found that food preferences were maintained for at least four days (Figure 2). This preference, and thus the memory of the pairing of the demonstrated odor with the social cue, persists even when the food is paired with concentrations of warfarin that will elicit significant distress. Although we predict that these preferences would have been maintained for even longer periods of time, longer timepoints were not tested as repeated exposure to the warfarin would have lead to unacceptable distress and death.

We found that food preferences induced by guanylin-family peptides were not noticeably impacted by the presence of a potentially dangerous food, containing the rodenticide warfarin, even when an unadulterated food alternative was available (Figure 3). This is consistent with the results of experiments in which observer rats do not acquire aversions to foods if the demonstrator rat was made ill (Galef et al., 1983). It has been suggested that rodents do not require socially transmitted taste avoidance since rodents, particularly rats, are neophobic and will generally avoid foods with a novel odor (Galef, 1985; Galef and Beck, 1985). This finding was recently confirmed by Jing et al. (2014), who found that observer rats acquired food preferences from anesthetized or partially anesthetized demonstrators that were made sick from LiCl injections. These authors suggest that rats do not lack the ability to detect the health status of conspecifics but instead lack the ability to detect potential danger from novel food. Therefore, at least in rodent species, it appears that negative outcomes regarding food are not socially transmitted. Conversely, social learning may be able to override learned avoidance. For example, rats will learn to avoid a dark chamber when entrance to that chamber is paired with foot shocks. This avoidance is inhibited if the experienced rat is paired with another rat in a “safe” chamber where no foot shocks are given (Masuda and Aou, 2009). Furthermore, devaluation of the odor used as a social cue (e.g., CS_2_) by pairing it with an aversive taste cue does not eliminate that odor's ability to elicit the acquisition of food preferences (Maier et al., 2014).

Mice, rats, and many other rodents are prevalent and costly pests. They damage crops, consume and contaminate human food and animal feed, damage infrastructure, and transmit human and animal diseases. The economic cost of damage by rats in the U.S. is estimated to exceed $20 billion, while worldwide food losses attributed to rats alone exceed $30 billion (Pimentel et al., 2000; Almeida et al., 2013). Because of this immense economic impact, over $1.3 billion is spent yearly on rodent control strategies, including rodenticide baits that contain lethal compounds such as. While in many cases rodenticides, including warfarin or similar anticoagulants, are an inexpensive and fairly efficient tool for pest rodent control, they have significant limitations. For example, many rodents exhibit significant neophobia, reducing the likelihood that a rodent will approach and consume baits (Baker et al., 2007). Also, some rodenticides must be consumed repeatedly over several days in order to reach lethal levels; if rodents become ill after the initial bait consumption, they may associate this feeling with the bait and not return for subsequent feedings (Baker et al., 2007). Unfortunately, the effectiveness of natural and biological attractants to aid the return of rodents to bait stations has been difficult to determine. For example, studies showing the use of natural odors (lemon and ginger) showed promise when rats were tested in enclosures, but failed to work in field studies (Witmer et al., 2008). Therefore, new strategies are needed to enhance the acceptance and ingestion of edible baits in a species-specific manner, thus increasing bait effectiveness and reducing the potential for baits to be ingested by non-target species. The ability of semiochemicals such as guanylin-family peptides to engage innate preference mechanisms in target rodents may offer an opportunity to safely and efficiently promote bait ingestion by these animals, thus reducing the huge economic and health costs associated with rodent pests.

### Conflict of interest statement

Steven D. Munger is an author of a PCT/US patent application describing the use of guanylin peptides to promote edible ingestion by rodents. Neither author receives or has received personal financial benefit (e.g., royalties) associated with this patent or these studies. Neither institution or laboratory has received any corporate or private funding related to these studies; these studies were entirely supported by the National Institutes of Health and the State of Maryland. The authors declare that the research was conducted in the absence of any commercial or financial relationships that could be construed as a potential conflict of interest.
